# Genetic Dissection of the Obesity Paradox in Carotid Atherosclerosis Using a Hyperlipidemic Mouse Cohort

**DOI:** 10.3390/ijms27198762

**Published:** 2026-09-30

**Authors:** Kiyan Parvaresh, Firas Dalloul, Mei-Hua Chen, Lisa J. Shi, Muhammad Sarfraz Ali, Hideyuki Torikai, Weibin Shi

**Affiliations:** 1 Department of Radiology and Medical Imaging, University of Virginia, Charlottesville, VA 22908, USA; kgp9vy@virginia.edu (K.P.); fee7001@gmail.com (F.D.); sjchen@email.com (M.-H.C.); lisajshi@utexas.edu (L.J.S.); sfj2zp@virginia.edu (M.S.A.); ht4az@uvahealth.org (H.T.); 2 Biochemistry & Molecular Genetics, University of Virginia, Charlottesville, VA 22908, USA

**Keywords:** atherosclerosis, carotid artery, obesity, quantitative trait locus, carotid intima-media thickness

## Abstract

Overweight and obese individuals often exhibit lower mortality rates or better prognoses than lean or normal-weight individuals with stroke and other diseases, a phenomenon called the “obesity paradox”. Carotid atherosclerosis is a major cause of ischemic stroke, and body weight serves as a reliable surrogate for adiposity in adult mice. Phenotypic and genetic connections of carotid atherosclerosis with body weight were evaluated in 299 F2 mice (154 females, 145 males) derived from BALB/cJ and LP/J *Apoe* knockout (*Apoe*^-/-^) mice. F2 mice were fed a Western diet for 12 weeks. Atherosclerotic lesion sizes in left carotid arteries, body weight, coat color, plasma lipids, glucose, small dense LDL ApoB, and malondialdehyde were measured, and 11,000 single nucleotide polymorphism (SNP) markers were genotyped. Carotid lesion sizes were inversely correlated with body weight in both sexes. Genome-wide scans identified two significant quantitative trait loci (QTLs) for carotid atherosclerosis on chromosomes (Chr) 6 and 15 in an additive sex model, and five QTLs on Chr 6, 7, 12, 13, and 15 in an interactive sex model. Adjusting for body weight variation downgraded Chr 15 QTL (*Cath5*) in both models, whereas other QTLs upgraded in the additive sex model and downgraded in the interactive sex model. Human syntenic region of *Cath5* associated with carotid intima-medial thickness (cIMT) and waist-to-hip ratio (WHR). These findings indicate that the obesity paradox in carotid atherosclerosis is partially driven by shared genetic components that exert opposing effects on adiposity and plaque development and act through sex-independent mechanisms.

## 1. Introduction

Stroke is the leading cause of adult disability and the fourth leading cause of death in the United States [1]. Ischemic stroke, which occurs when blood flow to the brain is obstructed, accounts for over 80% of all stroke cases, arising from diverse etiologies including cardioembolism, small-vessel occlusion, and large-artery atherosclerosis [2]. Among large-artery diseases, carotid artery atherosclerosis represents a major contributor to cerebral ischemia, where plaque development and destabilization impair blood flow or precipitate artery-to-artery thromboembolism [3]. Atherosclerosis is a complex trait influenced by both environmental and genetic factors, with heritability estimated at ~50% for carotid plaque and carotid intima-media thickness (cIMT) [4,5,6], two validated ultrasound surrogates of subclinical atherosclerosis. A recent meta-analysis of seven genome-wide association studies (GWAS) involving 131,000 individuals across diverse ancestries identified 59 independent loci for cIMT [7]. However, because the individual effect sizes of human GWAS-detected variants are typically small, pinpointing causal genes remains challenging. Consequently, parallel quantitative genetic approaches using animal models are essential to accelerate gene discovery in carotid atherosclerosis.

*Apoe* knockout (*Apoe*^-/-^) mice develop all phases of atherosclerotic lesions in large and medium-sized arteries, which are heavily influenced by diet and genetic backgrounds [8]. Using segregating F2 populations from *Apoe*^-/-^ mouse strains across various genetic backgrounds, we and others conduct quantitative trait locus (QTL) analysis for carotid atherosclerosis. To date, 20 significant QTLs for carotid atherosclerosis (*Cath1* through *Cath20*) have been identified from five independent crosses of *Apoe*^-/-^ strains [9,10,11,12,13]. Crucially, segregating F2 cohorts also enables the simultaneous dissection of phenotypic correlations and shared genetic architecture between distinct traits [14,15,16].

Obesity, characterized by excessive adiposity, is an established risk factor for stroke. However, clinical observations reveal that established stroke patients with higher body mass index (BMI) frequently correlates with lower mortality rates and improved long-term prognosis [17,18,19,20,21]. This phenomenon, termed the “obesity paradox”, often manifests as a U-shaped or inverse relationship, where normal-weight or mildly overweight individuals experience better outcomes than underweight or severely obese counterparts [22,23].

It remains intensely debated whether the post-stroke obesity paradox reflects genuine biological protection, such as elevated protective adipokines or greater metabolic reserves, or merely epidemiological and methodological constraints. The latter includes reverse causation (e.g., pre-stroke disease-induced weight loss or post-stroke catabolism/sarcopenia), selection/collider bias (evaluating mortality exclusively conditioned on having experienced a stroke event), residual confounding (e.g., age of stroke onset, treatment intensity), and the inherent inaccuracy of BMI as a surrogate for body fat composition versus lean muscle mass. Disentangling these mechanisms in human populations is difficult due to environmental and demographic confounders, dietary variations, and complex genetic heterogeneity. In contrast, mouse models allow for strict diet, age, and environmental control and genetic manipulation. Total body weight serves as a validated, highly reliable surrogate for adiposity in adult mice, enabling precise quantitative genetic mapping [16]. Moreover, hyperlipidemic *Apoe*^-/-^ mice recapitulate key elements of both metabolic dysfunction and plaque development, offering an ideal system to interrogate how body mass and atherosclerosis interact at the genome level. Here, we evaluated the phenotypic and genetic connections between body weight and carotid atherosclerosis in a segregating F2 cohort derived from BALB/cJ and LP/J *Apoe*^-/-^ mice, the two progenitor strains whose genomes have been sequenced to facilitate fine mapping and candidate gene prioritization. Through high-density SNP genotyping and quantitative trait mapping, we sought to determine whether shared genetic loci with opposing effects on body weight and plaque burden contribute to the biological mechanisms underlying the obesity paradox.

## 2. Results

### 2.1. Sex Difference in Carotid Atherosclerosis

**Figure 1:**
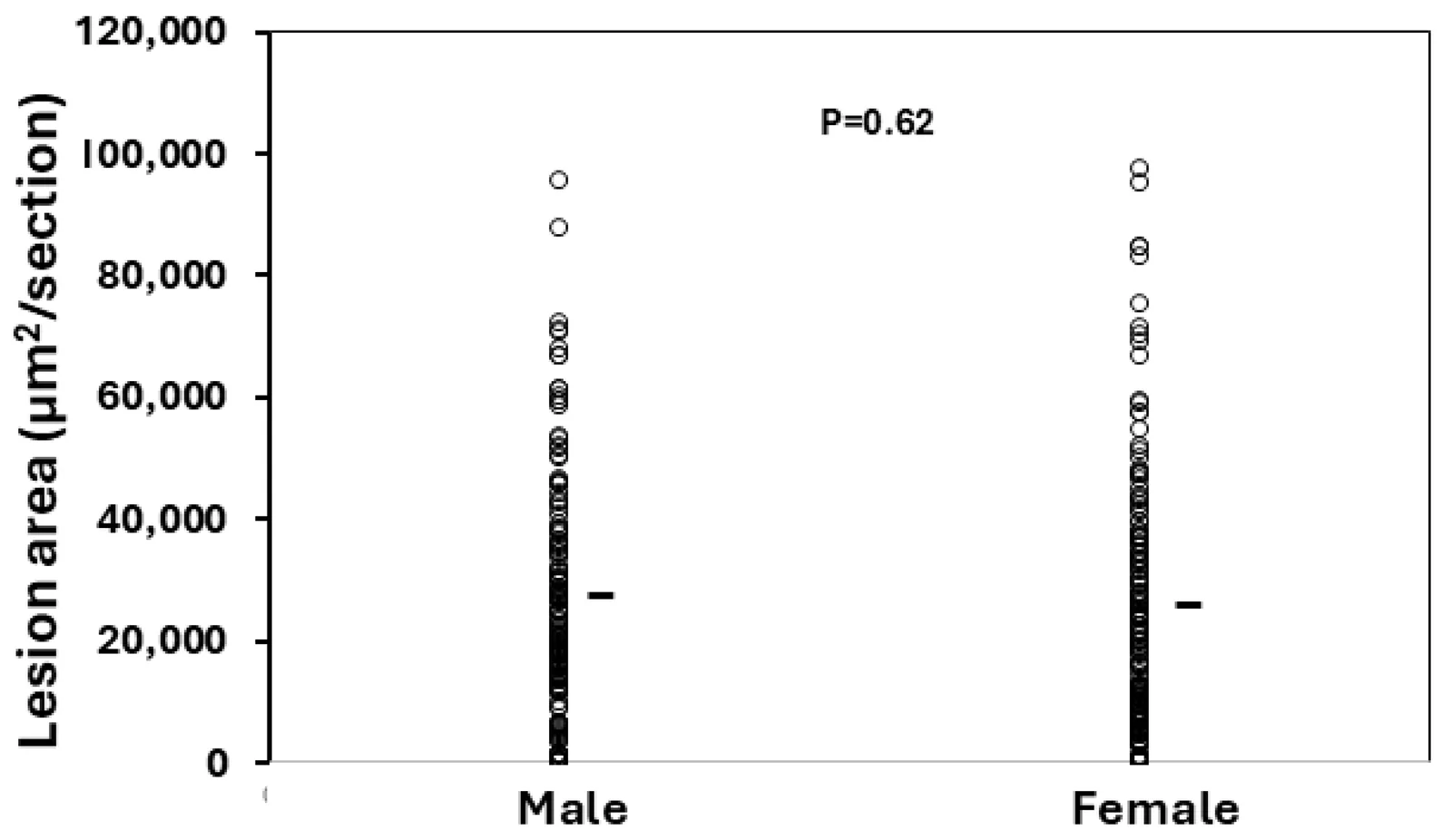
Atherosclerotic lesion sizes in the left carotid arteries of F2 mice derived from BALB/c- and LP-*Apoe*^-/-^ mice. Male and female F2 mice were fed a Western diet for 12 weeks. Each symbol represents an individual mouse. The short horizontal lines denote the mean for each group. The *p* value was calculated using a two-tailed Student’s T-test.

**Figure 2:**
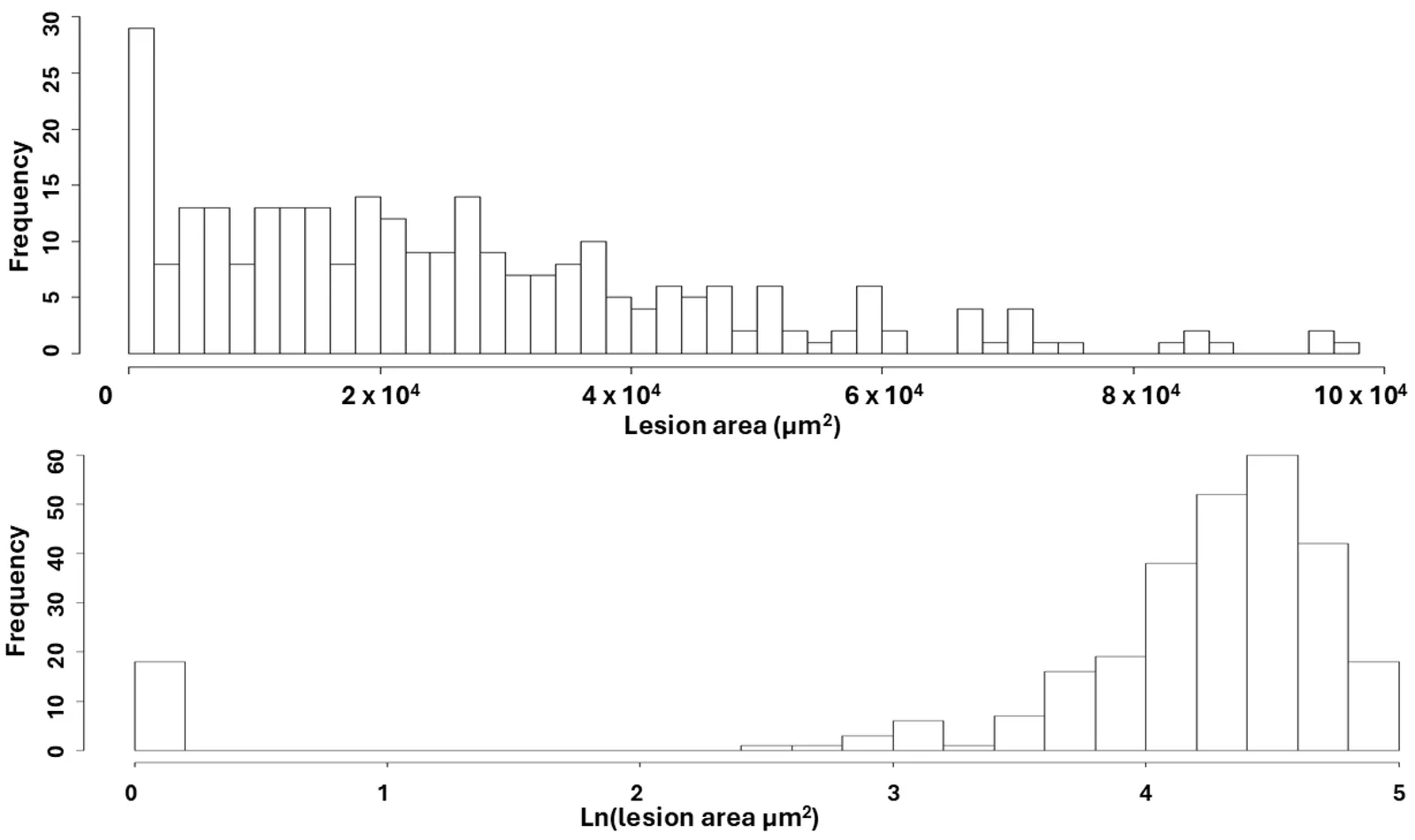
Distributions of untransformed and log-transformed carotid lesion sizes in both male and female F2 mice. Histograms showing untransformed (**top**) and natural log (Ln)-transformed (**bottom**) atherosclerotic lesion areas. Plots were generated using the plot distribution functions in R/qtl.

Atherosclerotic lesion sizes in the left carotid arteries of female and male F2 mice were measured after 12 weeks on the Western diet. Male mice developed slightly larger lesions than female F2 mice (26,426 ± 20,057 vs. 25,201 ± 21,775.8 µm^2^/section), though the difference was not statistically significant (*p* = 0.62) (Figure 1, Supplementary Materials). Lesion sizes varied widely among individual F2 mice, and 6% of the cohort developed no lesions (Figure 2). Log-transformed lesion sizes followed a bimodal distribution: a bar on the far left represents the lesion-free mice, while a bell-shaped histogram on the right tracks those that developed various sizes of carotid lesions.

### 2.2. QTL Analysis of Atherosclerosis

**Figure 3:**
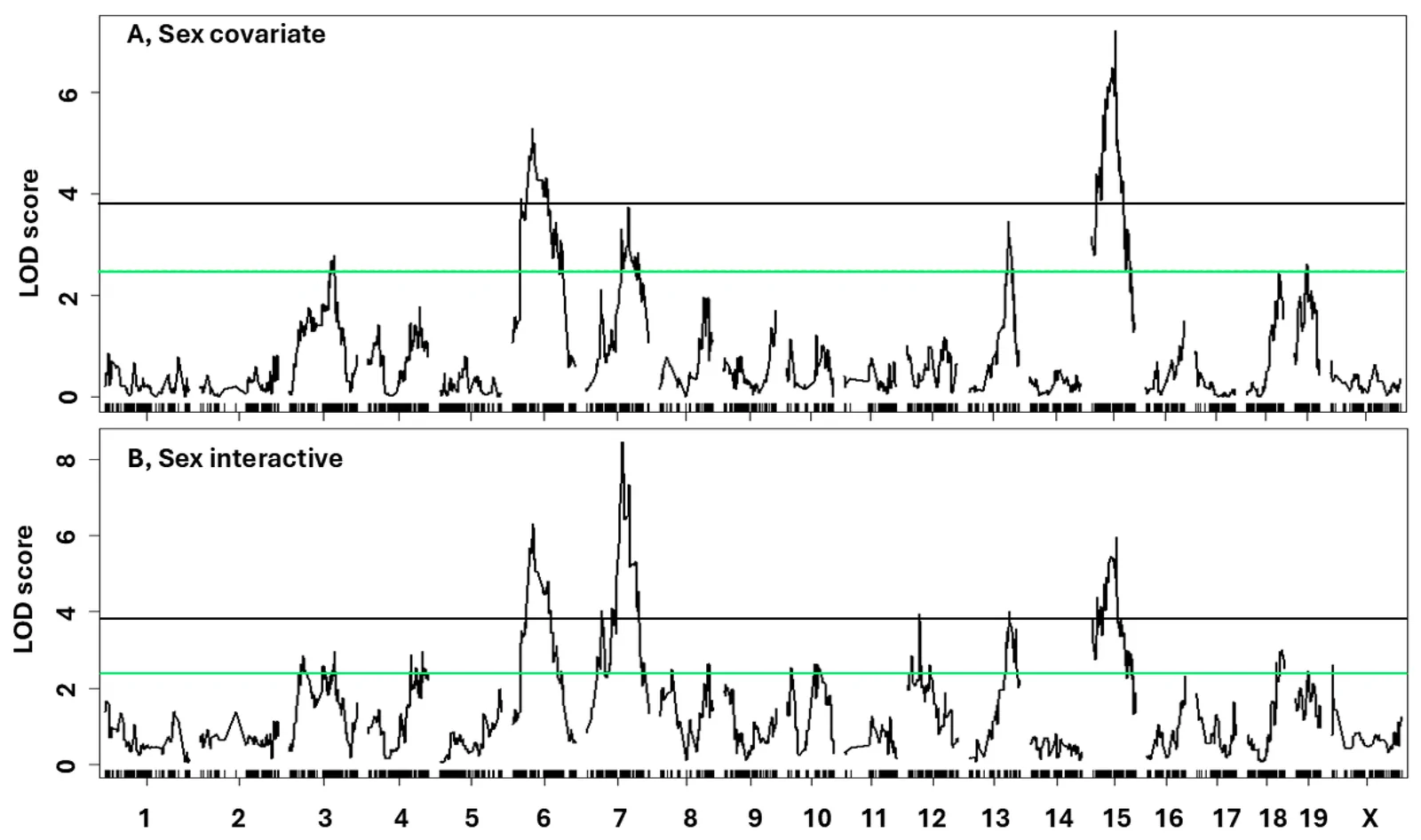
Genome-wide QTL scans for carotid atherosclerotic lesion sizes. (**A**), Genome-wide scan across all F2 mice using sex as a covariate. (**B**), Genome-wide scan across all F2 mice using sex as an interactive covariate. Chromosomes 1 through X are plotted along the X-axis, and the Y-axis indicates the LOD score. Horizontal green and black lines represent genome-wide thresholds for suggestive (*p* < 0.63) and significant (*p* < 0.05) linkage, respectively. Short vertical tick marks on the X-axis denote the chromosomal positions of genetic markers.

QTL analysis of the entire F2 cohort using sex as a covariate revealed two significant loci on chromosomes (Chr) 6 and 15, and four suggestive loci on Chr 3, 7, 13, and 19 for atherosclerotic lesion sizes (Figure 3A). Table 1 provides comprehensive details for these loci, including locus names, LOD scores, peak markers, % variance, 95% confidence intervals (CI), modes of inheritance, high-risk alleles, and allelic effects.

The Chr 6 QTL had a significant LOD score of 5.29 and peaked at 50.7 Mb. This QTL replicates *Cath4*, which was initially mapped in a BXH cross and subsequently replicated in a C57BL/6 x BALB *Apoe*^−^/^−^ cross [9,10]. The Chr 15 QTL at 57.3 Mb showed a significant LOD score of 7.22, replicating *Cath5*, previously mapped in BXH and BALB x SM *Apoe*^−^/^−^ intercrosses [11,24]. The Chr 7 QTL near 98.2 Mb and the Chr 13 QTL near 95.2 Mb had suggestive LOD scores of 3.75 and 3.46, respectively. The Chr 13 locus replicates *Cath3*, originally mapped in a C57BL/6 x BALB/c cross [10]. The suggestive QTLs on Chr 3 and Chr 19 are novel.

We also performed a genome-wide QTL scan for carotid lesions using sex as an interactive covariate (Figure 3B). This scan detected 5 significant QTLs on Chr 6, 7, 12, 13, and 15 and 7 suggestive QTLs on Chr 3, 4, 8, 10, 18, 19, and 20. Compared to the scan using sex as an additive covariate, the interactive sex scan revealed additional QTLs on Chr 8, 10, 12, 18, and 20. Among these, the Chr 12 QTL near 29.5 Mb was significant, with a LOD score of 3.94. This QTL replicates *Cath1*, which was initially mapped in a BXH cross and later verified in other crosses [10,11,24]. The remaining four QTLs are novel and showed suggestive LOD scores.

### 2.3. Associations of Carotid Atherosclerosis with Body Weight and Other Measures

Associations of carotid lesion sizes with body weight and other measures were determined with male, female, and entire F2 cohorts (Table 2, Supplementary Materials). Body weight was the only measure significantly associated with carotid lesion size in both male and female F2 mice, accounting for 5.2% of the variance in females and 4.5% in males (r^2^ values). Female F2 mice also showed significant inverse correlations with coat color intensity (r = −0.387, *p* = 1.6 × 10^−6^) and fasting plasma HDL cholesterol levels (r = −0.181, *p* = 0.03), which accounted for 15% and 3.3% of the variance in lesion sizes, respectively. In contrast, male F2 mice showed no association with these two traits or most other measures.

When all F2 mice were analyzed using a generalized linear model with sex as a covariate, carotid lesions were inversely associated with body weight (r = −0.14, *p* = 0.019) and coat color intensity (r = −0.25, *p* = 2.6 × 10^−5^) and positively associated with plasma malondialdehyde (r = 0.12, *p* = 0.047) and small dense LDL ApoB levels (r = 0.12, *p* = 0.046).

### 2.4. Causal Relationship Between Atherosclerosis and Body Weight

**Figure 4:**
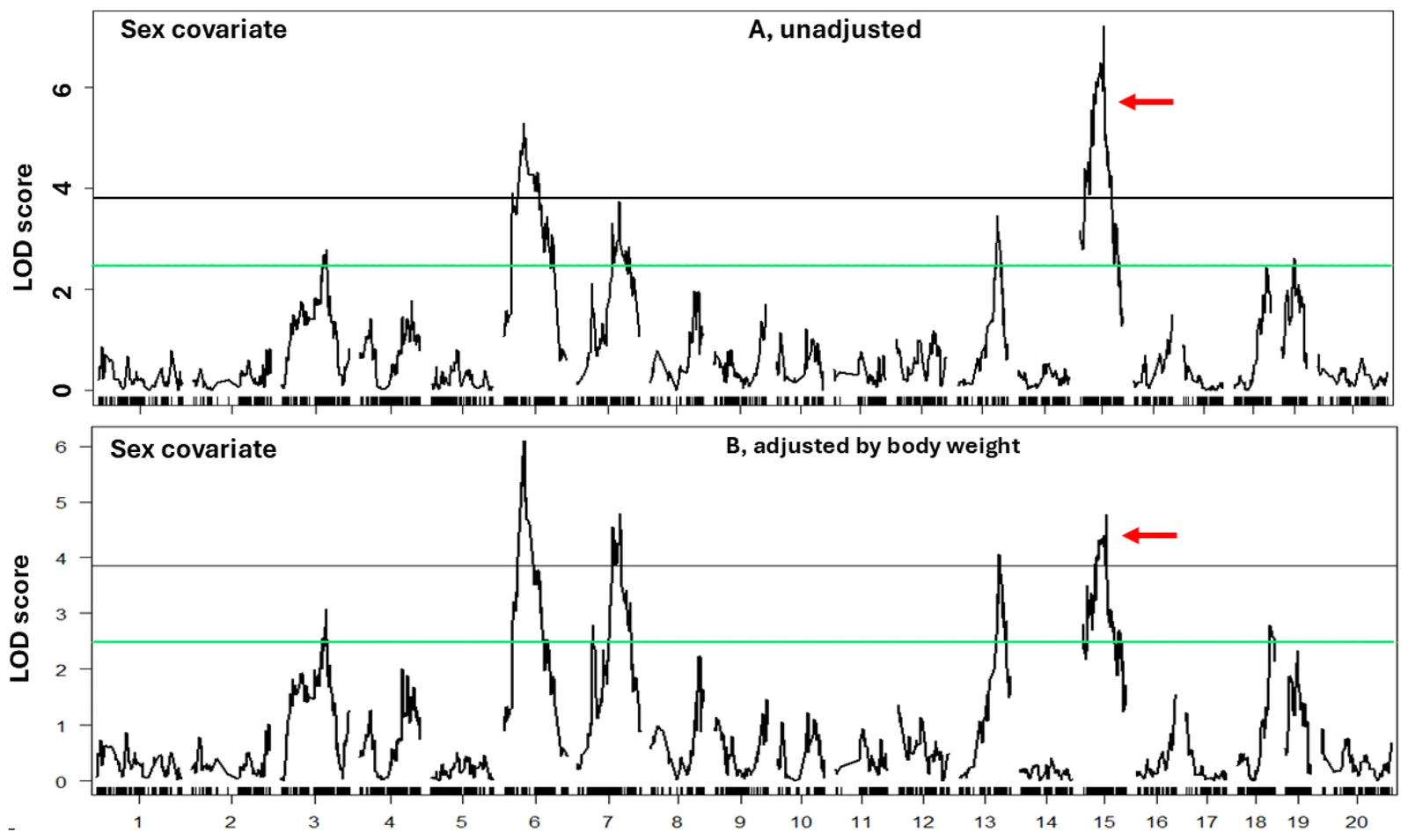
Genome-wide QTL analysis to evaluate the dependence of atherosclerosis QTLs on body weight by using sex as a covariate. (**A**), Genome-wide scan for atherosclerosis without adjusting for body weight. (**B**), Genome-wide scan for atherosclerosis after adjustment for body weight. Note the significant downgradation of chromosome 15 QTL (pointed by red arrow). The peak LOD scores of chromosomes 6, 7, and 13 QTLs showed moderate elevation.

**Figure 5:**
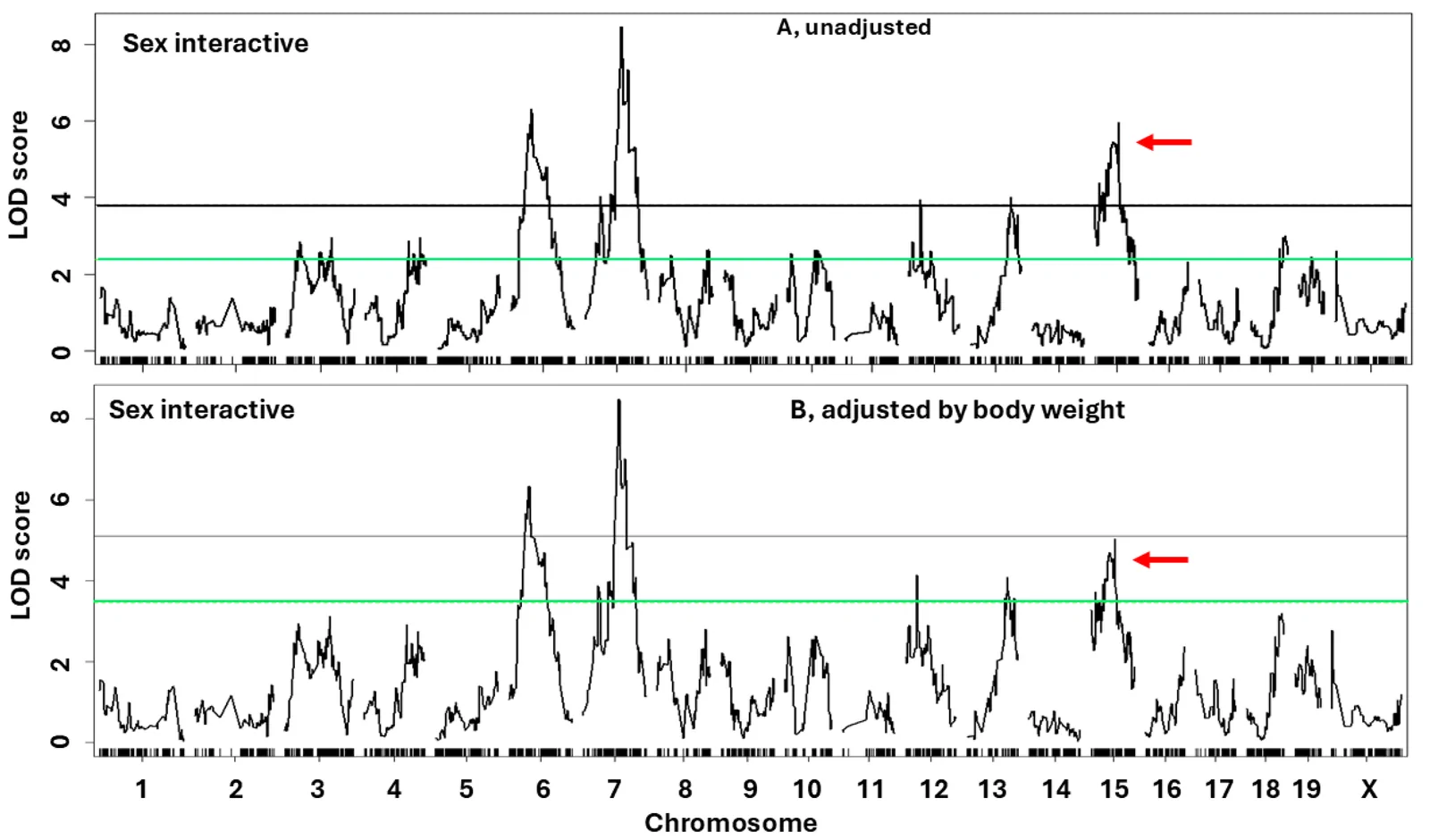
Genome-wide QTL analysis to evaluate the dependence of atherosclerosis QTLs on body weight by using sex as an interactive covariate. (**A**), Genome-wide scan for atherosclerosis without adjusting for body weight. (**B**), Genome-wide scan for atherosclerosis after adjusting for body weight. Note the downgradation of chromosome 15 QTL from significant to suggestive linkage (pointed by red arrow). Chromosomes 12 and 13 QTLs also downgraded from significant to suggestive linkage, but their LOD scores showed little change.

To evaluate the potential causal effect of body weight on carotid atherosclerosis, we performed a genome-wide QTL scan on the residuals obtained from a regression analysis of atherosclerotic lesion sizes against body weight in F2 mice. When genome-wide scan was conducted using sex as an additive covariate, the LOD score of the Chr 15 QTL decreased from 7.22 to 4.78, whereas the Chr 7 and Chr 13 QTLs were elevated from suggestive to significant linkage (Table 3, Figure 4). The QTLs on Chr 3, 6, and 18 remained largely unaffected by body weight adjustment. Furthermore, when the residuals were analyzed using sex as an interactive covariate, the Chr 15 QTL LOD score dropped from 5.95 to 4.78, while all other QTLs remained largely unchanged (Figure 5).

### 2.5. Candidate Genes for Chr 15 Atherosclerosis QTL

Within the *Cath5* confidence interval on Chr 15, 21 protein-coding genes contain one or more non-synonymous or upstream regulatory variants between the LP and BALB strains. Among these, *Snx31*, *Dcstamp*, *Klhl38*, and *Fer1l6* contain non-synonymous SNPs that cause amino acid substitutions (Table 4). Notably, BALB mice carry two predicted deleterious mutations with low SIFT scores of 0.02: an A/G SNP (rs249728855) in *Klhl38* causing an R1450C substitution, and a G/T SNP (rs216528441) in *Fer1l6* causing a G43C substitution.

Seventeen genes, including *Fbxo43*, *Polr2k*, *Ankrd46*, *Fzd6*, *Dcstamp*, *Dprs*, *Oxr1*, *Pkhd1l1*, *Tnfrsf11b*, *Ccn3*, *Deptor*, *Zhx2*, *Atad2*, *Ntaq1*, *Anxa13*, *Tmem65*, and *Mtss1*, contain one or more SNPs in their upstream regulatory regions, which may alter their protein expression levels.

### 2.6. Association with Carotid Atherosclerosis and Adiposity in Humans

**Figure 6:**
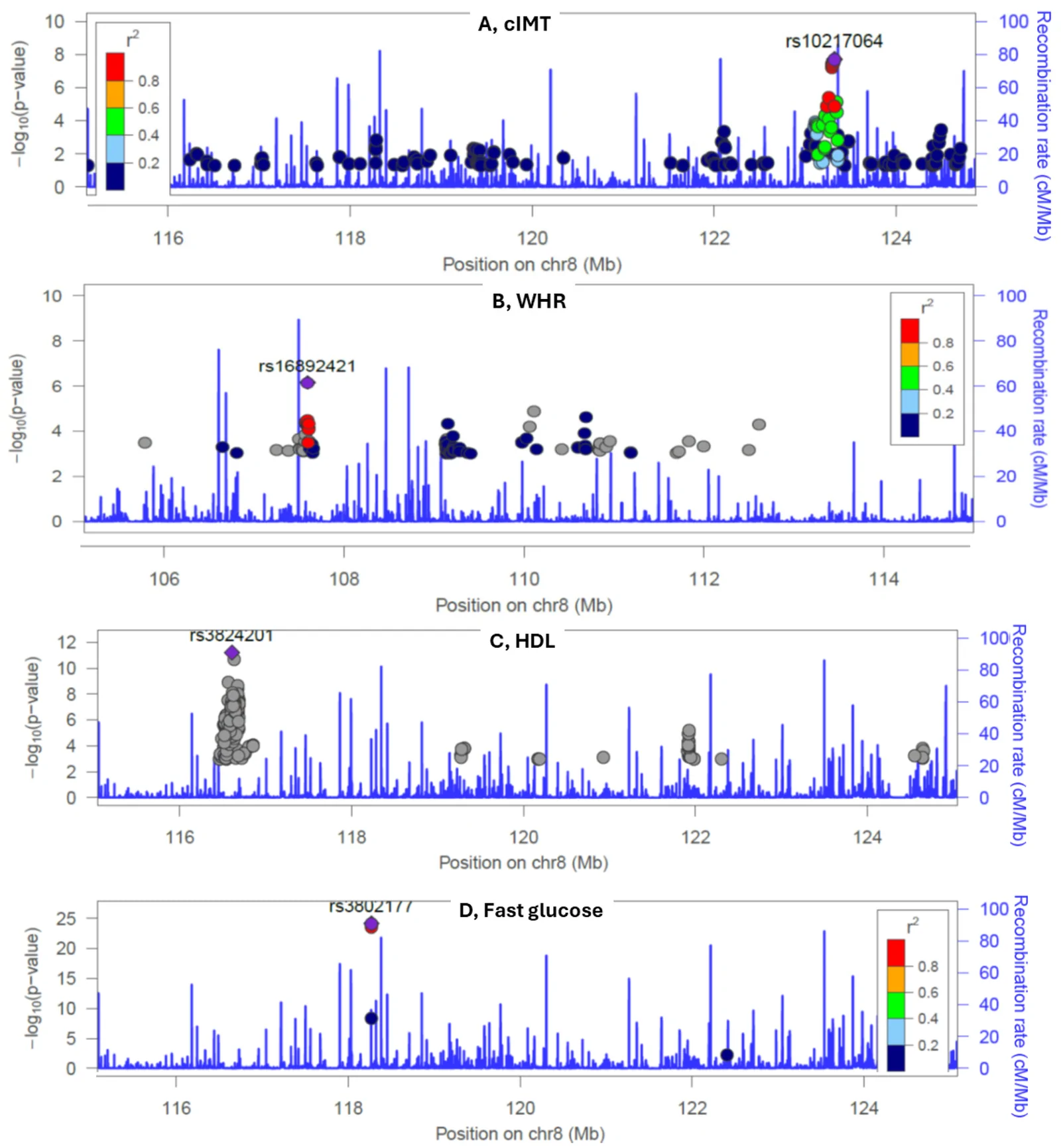
Regional association plots for human chromosome 8 (96–125 Mb), showing associations with carotid intima-media thickness (cIMT), waist-to-hip ratio (WHR), HDL cholesterol, and fasting glucose levels. GWAS meta-analysis dataset was derived from the CHARGE consortium. This genomic region corresponds to the confidence interval of *Cath5* on mouse chromosome 15. The purple dot represents the leading SNP in the selected region.

The confidence interval of *Cath5* extended from 35 to 59 Mb mouse chromosome 15, corresponding to syntenic regions on human chromosomes 5 (9–10.3 Mb) and 8 (96–125 Mb). Using GWAS meta-analysis data from the Cohorts for Heart and Aging Research in Genomic Epidemiology (CHARGE) consortium [25], we evaluated the associations of these syntenic regions with carotid intima-media thickness (cIMT) and adiposity traits. Regional association plots revealed that the Chromosome 8 syntenic region was significantly associated with cIMT (rs10217064, *p* = 1.93 × 10^−8^) and waist-to-hip ratio (WHR) (rs16892421, *p* = 6.94 × 10^−7^) (Figure 6A,B). Furthermore, this region demonstrated robust associations with HDL cholesterol (rs3824201, *p* = 6.35 × 10^−12^) and fasting glucose levels (rs3802177, *p* = 7.10 × 10^−25^) (Figure 6C,D).

## 3. Discussion

In this study, we observed an inverse correlation between carotid lesion size and body weight in both male and female F2 cohorts, supporting the existence of the “obesity paradox” in carotid atherosclerosis. Two significant QTLs on chromosomes 6 and 15 were identified for carotid atherosclerosis in the additive sex model and five significant QTLs on chromosomes 6, 7, 12, 13, and 15 were mapped in the interactive sex model. Adjusting for body weight variation downgraded the chromosome 15 QTL in both sex additive and interactive models, while other QTLs were upgraded in the additive sex model and downgraded in the sex interactive model. Additionally, the human orthologous region of *Cath5* on mouse chromosome 15 was associated with carotid intima-media thickness, a common measure of subclinical atherosclerosis, and adiposity.

A major finding of this study is the inverse correlation between body weight and carotid lesion size observed in both male and female F2 mice. To our knowledge, this is the first study to report an inverse correlation between carotid atherosclerosis and body weight in a mammalian model. Similar inverse relationships were observed in two of three additional F2 intercrosses tested, specifically BALB/c × C57BL/6 and BALB/c × SM/J, but not C3H × C57BL/6 (unpublished data). The aortic root is the standard site for quantifying mouse atherosclerosis. Inverse correlations between aortic lesion size and body weight or fat mass were observed in this and other cohorts reported [15,26,27]. Body weight serves as a robust surrogate marker for total body fat in adult mice, with correlation coefficients consistently exceeding r>0.90 [15]. Thus, these collective findings demonstrate the existence of the obesity paradox in mouse atherosclerosis, including the carotid vascular bed.

In this cross, two significant QTLs for body weight mapped to chromosome 7 (*Bw1n* or *Bdwtq*) and chromosome 15 (*Bsbob5*) [15], overlapping the carotid atherosclerosis loci *Cath20* and *Cath5* in the confidence interval, respectively. This colocalization provided a statistical means for defining causal relationship between the two traits. Conditioning on body weight attenuated the LOD score of *Cath5* on chromosome 15 in both additive and interactive sex models. Unlike *Cath5*, the remaining suggestive and significant QTLs upgraded in the additive sex model (which controls for main effects of sex) and downgraded in the interactive sex model (which allows the QTL effect to differ between males and females). These results indicate that the chromosome 15 QTL *Cath5* acts in a body weight-dependent but sex-independent manner on carotid atherosclerosis, whereas the remaining loci were affected by body weight through sex-dependent mechanisms. These results reveal that body weight variations previously acted as confounding variance, masking the primary effects of these loci on carotid atherosclerosis.

It is noteworthy that the downgrade of QTLs other than *Cath5* following body weight adjustment in the sex-interactive model resulted from the elevated permutation-derived significance threshold rather than reduced absolute LOD scores. Indeed, with the exception of *Cath5* on Chromosome 15, the LOD scores of all other loci remained essentially unchanged post-adjustment. Nevertheless, because of the more stringent statistical threshold, seven previously suggestive QTLs lost their significance. Taken together, these results suggest that *Cath5* acts through shared, opposing mechanisms on body weight and plaque burden, whereas the remaining QTLs operate independently of body weight via sex-influenced mechanisms. Indeed, incorporating sex as an interactive covariate unmasked additional loci on chromosomes 4, 8, 10, 12, 18, and 20. Significantly, the chromosome 12 QTL replicates *Cath1* mapped in BXH, B6 x BALB/c crosses [9,10,11,24]. The identification of additional loci across the genome underscores the power of accounting for sex-specific interactions in carotid atherosclerosis.

While male F2 mice exhibited slightly larger atherosclerotic lesions in the left carotid artery compared to females, this difference was not statistically significant. Notably, lesion burden varied widely across the cohort, with 6% of mice remaining completely resistant to lesion formation despite the extreme hypercholesterolemia developed during the 12-week Western diet feeding. This contrasts with observations at the aortic root, where all F2 mice developed atherosclerotic lesions [15]. Furthermore, female F2 mice developed significantly larger aortic root plaques than males in both the current cohort and previously crosses [15,26,28,29].

Carotid lesion sizes in female mice exhibited additional inverse correlations with both coat color intensity and fasting HDL cholesterol levels under Western diet feeding, whereas no such correlations were observed in males. *Tyr*, encoding tyrosinase, is the primary determinant of coat color in mice and represents a strong candidate gene for *Cath20* on chromosome 7. In BALB/cJ mice, a point mutation renders the tyrosinase protein nonfunctional. Tyrosinase catalyzes the oxidation of phenols (including L-tyrosine, L-DOPA, and dopaquinone) and is abundantly expressed in atherosclerotic lesions, particularly by vascular smooth muscle cells. Its inverse correlation with plaque sizes suggests a protective role in carotid atherosclerosis, although the mechanisms underlying this female-specific effect remain to be elucidated.

Across the entire cohort, carotid lesion sizes showed a weak but statistically significant correlation with plasma levels of malondialdehyde (MDA), a systemic oxidative stress marker, and small dense LDL ApoB concentrations. Other traditional risk factors, including non-HDL cholesterol, triglyceride, and glucose levels showed no association with carotid lesion sizes in the F2 mice. Poor or no associations of metabolic traits with carotid lesion sizes have been observed in other mouse crosses [9,10,11,24].

The mouse *Cath5* region maps syntenically to human chromosomes 5 (9–10.3 Mb) and 8 (96–125 Mb). The chromosome 8 syntenic locus has consistently associated with cIMT [30,31,32], although the exact genes involved remain unknown. Leveraging bioinformatic tools and resources, we prioritized *Klhl38* and *Fer1l6* as promising candidate genes for *Cath5* on chromosome 15. Both genes reside beneath the linkage peak and harbor one or more nonsynonymous coding variants that have a deleterious effect on protein function. *Klhl38*, encoding Kelch-like family member 38, and *Fer1l6*, encoding Fer-1-like family member 6, represent prime functional candidates involved in ubiquitin-proteasome signaling and membrane trafficking/repair in vascular or adipose tissues. Genome-wide association meta-analyses in over 100,000 human subjects revealed associations of *Klhl38*, *Zhx2*, and *Fbxo32* with cIMT [30]. *Zhx2* and *Fbxo32* are also located within the linkage peak of *Cath5* and contain upstream regulatory variants. *Zhx2* is abundantly expressed in both adipose and arterial tissues while *Fbxo32* is minimally expressed in either tissue. Other candidate genes, including *Tnfrsf11b*, *Fzd6*, *Ccn3*, *Deptor*, and *Oxr1*, contain upstream regulatory variants. *Tnfrsf11b* and *Ccn3* are of particular interest due to their involvement in vascular calcification, endothelial integrity, and smooth muscle cell proliferation. Serum Tnfrsf11b (Osteoprotegerin) levels strongly correlate with cIMT and metabolic syndrome in humans, making it a compelling candidate for the obesity paradox in atherosclerosis [33]. As a key inhibitor of mTORC1 and mTORC2 complexes, Deptor plays a critical cell-autonomous role in adipogenesis, nutrient sensing, and lipid accumulation, as well as endothelial cell activation.

The phenotypic overlap at *Cath5* could stem from three potential biological scenarios: First, true pleiotropy, where a single functional variant in a gene like *Deptor* or *Tnfrsf11b* independently regulates metabolic rate in adipose tissue and inflammatory responses in the carotid wall. Second, causal mediation, where the locus primarily regulates body weight/adiposity, which secondarily alters systemic adipokines or cytokines driving carotid plaque formation. Finally, close physical linkage, where two tightly linked distinct variants on Chr 15 separately control body weight and carotid lesion susceptibility. Functional studies with gene engineered mice are needed to pinpoint the underlying molecular mechanism.

Interestingly, the same human syntenic region showed associations with waist-to-hip ratio (WHR), HDL cholesterol, and fast blood glucose, all of which are risk factors for atherosclerosis. Thus, there is a possibility that genetic factors predisposing to obesity may modulate atherosclerosis susceptibility through their effects on HDL and glucose metabolism. Indeed, *Zhx2* was found to function as a regulator of key genes influencing lipoprotein metabolism [34].

This study has several limitations: First, the F2 mice were fed a Western diet and consequently developed severe hypercholesterolemia, which may mask or attenuate the impact of modest non-lipid cardiometabolic risk factors on carotid atherogenesis. On a standard chow diet, *Apoe*^-/-^ mice develop moderate hypercholesterolemia; consequently advanced carotid atherosclerosis typically requires many months to form [35]. ApoE is a multi-functional protein that regulates macrophage cholesterol efflux, dampen pro-inflammatory cytokine expression, suppress T-cell proliferation, and facilitate efferocytosis. In *Apoe*^-/-^ mice, the systemic absence of apoE creates a primed, hyper-inflammatory immunological microenvironment that accelerates plaque progression. Second, the inverse relationship observed between body weight and lesion size, the “obesity paradox” identified here, is restricted to subclinical plaque burden. Because carotid plaque is an upstream risk of ischemic stroke, this finding does not imply that higher body weight confers a protective effect on post-stroke clinical mortality, functional recovery, or long-term prognosis. Third, while body weight serves as a strong surrogate for total adiposity in adult mice, direct quantification of body composition (fat mass vs. lean mass via dual-energy X-ray absorptiometry or magnetic resonance imaging) provides necessary precision. Fourth, our QTL analysis focused on plaque burden rather than plaque stability, vulnerability features, or acute thrombotic occlusion, which was rarely observed in this F2 cohort. Under the 12-week Western diet regimen, most mice developed advanced atherosclerotic lesions exhibiting classic features such as fibrous caps, necrotic cores, calcification, cholesterol cleft, and acellular lipid deposits [36,37,38]. Finally, unlike human stroke patients who experience complex post-event lifestyle, metabolic, and environmental changes, our assessment was restricted to cross-sectional lesion measurements under tightly controlled experimental conditions of age, sex, and diet.

In summary, we have observed the paradoxical association of body weight, a surrogate indicator of fatness, with carotid atherosclerosis in a segregating F2 mouse population. The locus *Cath5* on Chr 15 represents a major pleiotropic genetic link between carotid atherosclerosis and body weight, operating through a sex-independent manner. The demonstration of genetic connections between the two traits provides an explanation for their paradoxical relationship. Functional annotation prioritizes *Klhl38*, *Fer1l6*, and regulatory vascular genes within *Cath5*. Further validation of these prioritized candidates will help elucidate the precise cellular mechanisms underlying the obesity paradox in carotid atherosclerosis.

## 4. Methods

### 4.1. Mice

Initially, 154 female and 145 F2 mice were generated from an intercross between male LP/J (LP) and female BALB/cJ (BALB) *Apoe*^-/-^ mice, as previously reported [16]. At 6 weeks of age, all F2 mice started a Western diet (TD 88137, Envigo, Indianapolis, IN, USA) for 12 weeks. Mice were housed under standard 12-h light/dark cycles with ad libitum access to food and water. Non-fasting blood samples were collected twice: once before the diet began and once after 11 weeks on the diet. At the end of the 12-week feeding period, body weight was measured and fasting blood samples were collected following an overnight fast. All blood samples were taken from the retro-orbital plexus of mice under isoflurane anesthesia, and plasma was prepared as described previously [39]. All procedures were approved by the Institutional Animal Care and Use Committee (protocol #: 3109).

### 4.2. Phenotypic Analyses

Atherosclerotic lesion sizes in the left carotid artery of F2 mice were measured by the first two coauthors who were blinded to the genotype, sex, and body weight of mice. As previously reported [9], the vasculature of mice was perfusion-fixed with 10% formalin via the left ventricle. The distal portion of the common carotid artery and adjacent branches were dissected and embedded in Tissue-Tek optimum cutting (OCT) compound. 10-μm-thick cryosections were collected every three sections and stained with oil red O and hematoxylin. Lesion sizes were quantified using Zen imaging software, version 3.14. For each mouse, the mean lesion area was calculated from the five sections exhibiting the largest lesions and this mean value was used for statistical analysis.

Plasma concentrations of cholesterol and triglycerides were measured using enzymatic assays as reported [39]. HDL cholesterol was measured after precipitation of other lipoproteins using a phosphotungstate-magnesium reagent (Wako Diagnostics, Richmond, VA, USA). Non-HDL cholesterol was calculated by subtracting HDL cholesterol from total cholesterol concentrations. Plasma glucose was measured using a Sigma assay kit (Cat. # GAHK20, St. Louis, MI, USA) as reported [39]. Plasma small dense LDL was determined by first precipitating non-small dense LDL, IDL, VLDL lipoproteins, and chylomicrons using a phosphotungstate-magnesium reagent (FUJIFILM Wako, Richmond, VA, USA) and then measuring the remaining ApoB concentration using an ELISA kit (MyBiosource, San Diego, CA, USA; Cat. #: MBS937790) [40]. Plasma levels of malondialdehyde, an indicator of lipid peroxidation, were measured using a Cayman Thiobarbituric Acid Reactive Substances assay kit (TBARS) kit (Cayman Chemical, Ann Arbor, MI, USA. Cat. # 10009055).

### 4.3. Genotypic Analysis

DNA was extracted from tail clips and genotyped at Neogen (Lansing, MI, USA) using miniMUGA arrays containing 11,000 SNP probes. DNA from parental strains and F1 hybrids was included on each array as controls. SNP markers were excluded if they showed discordant genotypes for control samples or deviated significantly from the Hardy–Weinberg equilibrium. Potential genotyping errors were further checked using the “calc errorlod” function in the R/qtl package. After filtration, 2594 informative SNPs remained for QTL analysis.

### 4.4. Statistical Analysis

QTL analysis was performed using the R/qtl package as previously described [41]. Genome-wide thresholds for significant (*p* < 0.05) and suggestive (*p* < 0.63) linkage were determined by 1000 permutations using nonparametric and expectation-maximization (EM) algorithms across the genome [42,43]. Because many traits differed significantly between male and female mice, QTL scans were performed using all F2 mice of both sexes with sex included as either an additive or an interactive covariate to maximize statistical power.

To model sex effects, we implemented two standard linear regression models across genome-wide scans:

Additive Sex Model assumes that sex affects the phenotype value identically across all genotypes without altering the genotype effect size:

$$y_{i}=\mu +\;\beta_{g}g_{i}+\;\beta_{s}s_{i}+\;\epsilon_{i}$$

where $y_{i}$ is the phenotype (carotid lesion area) for mouse  i, μ is the overall mean, $g_{i}$ is the additive genotype code, $s_{i}$ is sex (0 = male, 1 = female), $\beta_{g}$ and $\beta_{s}$ are main effects, and $\epsilon_{i}$ is the residual error.

Interactive Sex Model allows for sex-by-genotype interaction, permitting the QTL effect size to differ between males and females:

$$y_{i}=\mu +\;\beta_{g}g_{i}+\;\beta_{s}s_{i}+\;\gamma_{g\;\times \;s}\left(g_{i}\times \;s_{i}\right)+\;\epsilon_{i}$$

where $\gamma_{g\;\times \;s}$ represents the interaction coefficient between genotype and sex.

QTLs with a genome-wide *p* value < 0.05 were significant, and those with a genome-wide *p* value < 0.63 were suggestive.

The Student *t* test was used to compare quantitative traits between male and female F2 mice, and linear regression was used to assess associations between two continuous variables. For traits that differed significantly by sex, the linear regression models were adjusted for sex. Statistical significance was defined as *p* < 0.05.

### 4.5. Causal Inference for Atherosclerosis and Body Weight

Potential causal relationships between atherosclerosis and body weight were evaluated using a regression-based conditioning method [44]. Briefly, residuals were generated from the linear regression of one trait against the other and then subjected to a genome-wide QTL scan using the same mapping algorithm. If one trait causally drives the other, conditioning on the causal mediator will eliminate or significantly reduce the LOD score of one or more QTLs for the dependent trait, rendering the residual variation independent of the driver trait.

### 4.6. Candidate Gene Prioritization

Bioinformatic resources were used to prioritize candidate genes for the chromosome 15 (Chr 15) atherosclerosis QTL that demonstrated genetic connections with body weight. Positional candidate genes were identified based on the presence of one or more nonsynonymous coding variants or variants within upstream regulatory regions (2 kb upstream of the transcription start site) between the LP and BALB progenitor strains. Strain-specific variant analysis was conducted using public databases, including the Mouse Phenome Database and Ensembl. The functional impact of nonsynonymous variants on protein function was predicted using SIFT (Sorting Intolerant From Tolerant) scores.

### 4.7. Association with Carotid Atherosclerosis and Adiposity in Humans

The confidence interval of mouse *Cath5* (35–59 Mb on Chr 15) maps syntenically to human chromosome 8 (96–125 Mb) and chromosome 5 (9–10.3 Mb). To evaluate the translational relevance of this region to human carotid atherosclerosis and obesity, we examined summary statistics from large-scale genome-wide association study (GWAS) meta-analyses conducted by the Cohorts for Heart and Aging Research in Genomic Epidemiology (CHARGE) consortium [31]. Although CHARGE initially comprised five founding cohorts: the Age, Gene/Environment Susceptibility–Reykjavik Study (AGES), Atherosclerosis Risk in Communities (ARIC) study, Cardiovascular Health Study (CHS), Framingham Heart Study (FHS), and Rotterdam Study, it has since incorporated genomic and phenotypic data from many additional cohorts.

Within each contributing study, traits were regressed on covariates to derive residuals, which were then inverse-normally transformed to achieve a standard normal distribution. For studies of unrelated individuals, each SNP was tested under an additive model by regressing transformed residuals against effect allele dosage. For studies with related individuals, family-based association testing was performed. Analyses were conducted in sex-combined samples, and results across studies were combined using inverse-variance weighted fixed-effects meta-analysis based on 20.5M SNPs imputed to the 1000 Genomes Project (Phase 1 v3) reference panel. Genome-wide significance was set at *p* < 5 × 10^−8^. Association statistics for cIMT and carotid plaque, measured via high-resolution B-mode ultrasonography, were evaluated in up to 46,271 participants across diverse ancestries [32], while waist-to-hip ratio (WHR) data were evaluated in 20,615 participants.

## Figures and Tables

**Table 1:** Suggestive and significant QTLs for carotid atherosclerosis mapped in F2 mice derived from LP and BALB *Apoe*^-/-^ mice.

Locus Name	Chr	LOD	Peak (Mb)	Closest Marker	% Var	95%CI (Mb)	High Allele	Mode of Inheritance	Female			Male		
									Allelic Effect	Allelic Effect	Allelic Effect	Allelic Effect	Allelic Effect	Allelic Effect
									BB	H	LL	BB	H	LL
**Sex as an additive covariate**														
-	3	2.8	107.2	mUNC030678789	4.5	26.3–125.6	B	Additive/Recessive	32,436 ± 24,209	24,182 ± 21,190	20,428 ± 19,390	34,792 ± 21,992	24,612 ± 19,884	23,361 ± 16,219
***Cath4***	**6**	**5.29**	**50.7**	**gUNC11063030**	**9.4**	**25.7–85.9**	**L**	**Recessive/additive**	**20,868 ± 21,035**	**22,598 ± 17,420**	**35,100 ± 26,909**	**18,627 ± 19,116**	**25,381 ± 17,628**	**40,502 ± 21,344**
*Cath20*	7	3.75	98.2	SBJ073928051	8.2	80.4–133.1	B (F), L (M)	Additive/Recessive	39,448 ± 26,483	20,556 ± 16,150	16,139 ± 14,606	25,509 ± 21,205	22,228 ± 16,019	35,858 ± 23,504
Cath3	13	3.46	95.2	SBJ133808455	6.3	89.1–109.6	L	Recessive	25,090 ± 20,018	20,565 ± 20,394	33,731 ± 23,780	25,509 ± 21,204	22,228 ± 16,019	35,858 ± 23,504
***Cath5***	**15**	**7.22**	**57.3**	**gUNC25658310**	**9.0**	**35.1–59.0**	**L**	Additive	**16,917 ± 21,544**	**26,512 ± 19,480**	**30,634 ± 23,919**	**13,805 ± 13,652**	**27,306 ± 19,074**	**34,455 ± 21,065**
-	19	2.61	31.6	gUNCHS047573	3.7	11.3–51.8	B	Additive	30,520 ± 22,552	24,453 ± 21,023	21,297 ± 22,437	34,643 ± 21,880	26,186 ± 19,514	20,800 ± 17,853
**Sex as an interactive covariate**														
-	3	2.97	107.2	mUNC030678789	4.5	25.5–159.1	B	Additive/Recessive	32,436 ± 24,209	24,182 ± 21,190	20,428 ± 19,390	34,792 ± 21,992	24,612 ± 19,884	23,361 ± 16,219
-	4	2.96	142.1	SSH045683570	3.1	32.3–156.1	B (F)	Additive	32,495 ± 22,080	26,540 ± 24,178	16,258 ± 11,717	30,445 ± 24,378	23,866 ± 19,021	26,555 ± 15,953
***Cath4***	**6**	**6.31**	**50.7**	**gUNC11063030**	**9.4**	**44.1–86.0**	**L**	**Recessive/Additive**	**20,868 ± 21,035**	**22,598 ± 17,420**	**35,100 ± 26,909**	**18,627 ± 19,116**	**25,381 ± 17,628**	**40,502 ± 21,344**
***Cath20***	**7**	**8.46**	**83.2**	**SX1073328646**	**7.7**	**80.4–98.2**	B (F)	Recessive	**42,252 ± 26,102**	**18,680 ± 16,291**	**18,784 ± 15,430**	27,592 ± 22,808	26,405 ± 18,965	25,268 ± 19,873
-	8	2.63	117.5	gJAX00681656	3.5	8.2–128.5	B	Dominant	30,502 ± 25,594	28,253 ± 21,711	17,497 ± 17,347	24,134 ± 16,822	29,269 ± 22,830	22,672 ± 16,835
-	10	2.63	85.6	UNC18355681	1.2	23.6–120.3	B (F)	Recessive	35,526 ± 27,529	21,289 ± 18,306	22,421 ± 17,308	24,347 ± 22,668	27,638 ± 20,186	26,506 ± 16,247
***Cath1***	**12**	**3.94**	**29.5**	**mUNC20806291**	**1.3**	**4.2–58.2**	**L (F), H (M)**	**Recessive/Heterosis**	**21,926 ± 18,442**	**21,335 ± 18,618**	**35,893 ± 26,919**	**23,675 ± 19,444**	**30,166 ± 20,094**	**21,805 ± 19,837**
***Cath3***	**13**	**4.01**	**95.2**	**SBJ133808455**	**6.3**	**89.1–112.5**	L	Recessive	25,090 ± 20,018	20,565 ± 20,394	33,731 ± 23,780	25,509 ± 21,204	22,228 ± 16,019	35,858 ± 23,504
***Cath5***	**15**	**5.95**	**57.3**	**gUNC25658310**	**9.0**	**30.3–58.1**	**L**	Additive	**16,917 ± 21,544**	**26,512 ± 19,480**	**30,634 ± 23,919**	**13,805 ± 13,652**	**27,306 ± 19,074**	**34,455 ± 21,065**
-	18	3	84.1	gUNC29747959	3.9	69.4–89.5	B	Dominant/Additive	25,534 ± 18,806	27,951 ± 25,189	17,816 ± 12,214	33,232 ± 22,330	25,892 ± 19,483	19,230 ± 15,437
-	19	2.45	31.6	gUNCHS047573	3.7	3.6–57.7	**B**	Additive	30,520 ± 22,552	24,453 ± 21,023	21,297 ± 22,438	34,643 ± 21,880	26,186 ± 19,514	20,800 ± 17,853
-	20	2.6	11.2	SXX200448070	1.3	9.8–168.8	B (F)	-	25,936 ± 21,266	21,290 ± 18,673	-	25,855 ± 20,520	-	26,915 ± 19,999

Significant QTLs and LOD scores were highlighted in bold. Chr, chromosome; CI, confidence interval; % Var, percent phenotypic variance explained by that QTL. BB: homozygous BALB allele; LL: homozygous LP allele; H: Heterozygous for both BALB and LP alleles. Unit for atherosclerotic lesion: µm^2^/section. Values for allelic effect were expressed as means ± SD. LOD thresholds for significant or suggestive linkage were determined through 1000 permutations. When sex served as a covariate, the significant and suggestive LOD thresholds were 3.82 and 2.49, respectively. When sex served as an interactive variant, the significant and suggestive LOD thresholds were 3.80 and 2.39, respectively. “-“ unnamed suggestive QTL.

**Table 2:** Association of carotid lesion sizes with risk factors in F2 mice.

Trait	R (♂ + ♀)	P (♂ + ♀)	R (♀)	P (♀)	R (♂)	P (♂)
Body weight	**−0.140**	**0.019**	**−0.228**	**0.006**	**−0.212**	**0.013**
Coat color	**−0.250**	**2.60 × 10^−5^**	**−0.387**	**1.56 × 10^−6^**	−0.089	0.308
LDL (chow, nonfast)	0.067	0.261	0.138	0.098	0.014	0.874
HDL (chow, nonfast)	0.091	0.126	0.110	0.187	0.063	0.465
Triglyceride (chow, nonfast)	0.076	0.204	0.059	0.482	0.094	0.277
Glucose (chow, nonfast)	0.041	0.489	0.088	0.290	0.012	0.888
LDL (Western, nonfast)	0.035	0.563	0.035	0.675	0.099	0.251
HDL (Western, nonfast)	0.010	0.863	−0.150	0.071	0.127	0.138
Triglyceride (Western, nonfast)	0.033	0.587	0.129	0.122	0.012	0.891
Glucose (Western, nonfast)	0.034	0.572	−0.145	0.082	0.163	0.058
LDL (Western, fast)	0.057	0.342	0.127	0.128	0.007	0.933
HDL (Western, fast)	0.039	0.514	**−0.181**	**0.030**	0.032	0.712
Triglyceride (Western, fast)	0.006	0.924	0.065	0.448	−0.062	0.479
Glucose (Western, fast)	0.100	0.095	0.105	0.095	0.099	0.253
MDA	**0.119**	**0.047**	0.127	0.129	0.104	0.225
Small dense LDL ApoB	**0.119**	**0.046**	0.095	0.259	0.139	0.104

Associations of atherosclerotic lesions with various risk factors were determined with generalized linear model adjusted by sex when both sexes of F2 mice were analyzed. Significant associations are highlighted in **Bold**. ♂ indicates male, ♀ female; significant correlations are highlighted in bold. Unit: body weight: gram; coat color graded into white (0), light brown (1), dark brown (2), and agoudi (3); plasma lipid and glucose levels: mg/dL; MDA (malondialdehyde): µM; small dense LDL in ApoB content: µg/mL.

**Table 3:** Suggestive and significant QTLs for carotid atherosclerosis after adjusting variance in body weight.

Locus Name	Chr	LOD	Peak (Mb)	Closest Marker	95%CI (Mb)
**Nonparametric, Sex covariate**					
-	3	3.08	107.2	mUNC030678789	-
***Cath4***	**6**	**6.10**	**50.7**	**gUNC11063030**	**44.1–61.8**
***Cath20***	**7**	**4.79**	**98.2**	**SBJ073928051**	**79.1–114.9**
**Cath3**	**13**	**4.05**	**95.2**	**SBJ133808455**	**89.1–110.7**
***Cath5***	**15**	**4.78**	**57.3**	**gUNC25658310**	**14.2–68.0**
gUNC29665086	18	2.79	78.7	gUNC29665086	-
**Nonparametric, Sex interactive**					
***Cath4***	**6**	**6.33**	**50.7**	**gUNC11063030**	**44.1–86.0**
***Cath20***	**7**	**8.50**	**83.2**	**mJAX00153722**	**80.4–98.2**
*Cath1*	12	4.13	29.5	mUNC20806291	-
*Cath3*	13	4.08	95.2	SBJ133808455	-
*Cath5*	15	5.03	57.3	gUNC25658310	-

Significant QTLs and LOD scores were highlighted in bold. Chr, chromosome; CI, confidence interval. When sex served as a covariate, the significant and suggestive LOD thresholds were 3.86 and 2.89, respectively. When sex served as an interactive covariate, the significant and suggestive LOD thresholds were 5.1 and 3.45, respectively.

**Table 4:** Candidate genes for *Cath5* on chromosome 15 (35–59 Mb).

Chr	Position (bp)	Gene	dbSNP	Reference Allele	LP Allele	BALB Allele	Consequence	Amino Acid Change	SIFT Score
15	36525824	Snx31	rs254563995	T	C	T	Coding-NonSynonymous	I/V	1
15	36537680	Snx31	rs243336997	T	C	T	Coding-NonSynonymous	Q/R	0.78
15	39618259	Dcstamp	rs249697335	A	G	A	Coding-NonSynonymous	I/V	1
**15**	**58179636**	**Klhl38**	**rs249728855**	**G**	**G**	**A**	**Coding-NonSynonymous**	**R/C (1450)**	**0.02**
**15**	**58420215**	**Fer1l6**	**rs216528441**	**G**	**G**	**T**	**Coding-NonSynonymous**	**G/C (43)**	**0.02**
15	36150656	Fbxo43	rs50864269	A	C	A	mRNA-UTR		
15	36151057	Fbxo43	rs256832564	C	T	C	mRNA-UTR		
15	36151058	Fbxo43	rs234740378	A	G	A	mRNA-UTR		
15	36151062	Fbxo43	rs253707387	G	A	G	mRNA-UTR		
15	36151076	Fbxo43	rs579911191	C	A	C	mRNA-UTR		
15	36151080	Fbxo43	rs213623065	T	C	T	mRNA-UTR		
15	36151081	Fbxo43	rs234948605	G	C	G	mRNA-UTR		
15	36151095	Fbxo43	rs50544453	A	G	A	mRNA-UTR		
15	36173459	Polr2k	rs253531154	C	A	C	697 bp upstream		
15	36173505	Polr2k	rs222235651	T	C	T	651 bp upstream		
15	36173556	Polr2k	rs236793457	C	T	C	600 bp upstream		
15	36173581	Polr2k	rs223378037	A	T	A	575 bp upstream		
15	36173608	Polr2k	rs243319721	G	A	G	548 bp upstream		
15	36173610	Polr2k	rs265050323	G	A	G	546 bp upstream		
15	36173648	Polr2k	rs232248714	T	C	T	508 bp upstream		
15	36173658	Polr2k	rs261252743	G	A	G	498 bp upstream		
15	36173674	Polr2k	rs228516136	A	G	A	482 bp upstream		
15	36173687	Polr2k	rs211724603	C	A	C	469 bp upstream		
15	36173893	Polr2k	rs253412103	C	T	C	263 bp upstream		
15	36173920	Polr2k	rs231034447	G	A	G	236 bp upstream		
15	36174065	Polr2k	rs46339956	A	C	A	91 bp upstream		
15	36174105	Polr2k	rs51527254	C	G	C	51 bp upstream		
15	36174117	Polr2k	rs51377649	G	A	G	39 bp upstream		
15	36479008	Ankrd46	rs248876101	A	G	A	mRNA-UTR		
15	38870723	Fzd6	rs217229554	G	A	G	mRNA-UTR		
15	39607998	Dcstamp	rs247820719	A	C	A	1328 bp upstream		
15	39608677	Dcstamp	rs224779800	G	T	G	649 bp upstream		
15	39608686	Dcstamp	rs249185052	T	C	T	640 bp upstream		
15	39608713	Dcstamp	rs215332624	C	T	C	613 bp upstream		
15	39608761	Dcstamp	rs251051142	G	T	G	565 bp upstream		
15	39608788	Dcstamp	rs252789426	A	G	A	538 bp upstream		
15	39632173	Dpys	rs248840711	C	T	C	mRNA-UTR		
15	39632174	Dpys	rs211930045	A	G	A	mRNA-UTR		
15	39632247	Dpys	rs257035289	C	G	C	mRNA-UTR		
15	39632388	Dpys	rs227987779	C	A	C	mRNA-UTR		
15	41310794	Oxr1	rs219089402	A	T	A	mRNA-UTR		
15	41310828	Oxr1	rs239148372	A	G	A	mRNA-UTR		
15	41310910	Oxr1	rs258614241	G	A	G	mRNA-UTR		
15	44320930	Pkhd1l1	rs239874708	C	G	C	mRNA-UTR		
15	44320931	Pkhd1l1	rs259180475	C	T	C	mRNA-UTR		
15	44320935	Pkhd1l1	rs217867183	G	A	G	mRNA-UTR		
15	54142899	Tnfrsf11b	rs215791616	T	C	T	1199 bp upstream		
15	54142900	Tnfrsf11b	rs230607506	T	C	T	1200 bp upstream		
15	54142901	Tnfrsf11b	rs255271314	T	A	T	1201 bp upstream		
15	54608918	Ccn3	rs50628029	A	G	A	388 bp upstream		
15	55118860	Deptor	rs222806293	A	G	A	mRNA-UTR		
15	55118970	Deptor	rs52292696	C	T	C	mRNA-UTR		
15	55119225	Deptor	rs585753441	C	G	C	mRNA-UTR		
15	55119227	Deptor	rs387492581	C	G	C	mRNA-UTR		
15	55119229	Deptor	rs386912942	C	A	C	mRNA-UTR		
15	55119402	Deptor	rs247603601	A	C	A	mRNA-UTR		
15	57703025	Zhx2	rs250230111	C	T	C	mRNA-UTR		
15	57999408	Atad2	rs225606221	G	G	T	930 bp upstream		
15	57999408	Atad2	rs225606221	G	G	T	930 bp upstream		
15	57999433	Atad2	rs251685732	C	C	G	955 bp upstream		
15	57999433	Atad2	rs251685732	C	C	G	955 bp upstream		
15	57999458	Atad2	rs235542084	G	G	A	980 bp upstream		
15	57999458	Atad2	rs235542084	G	G	A	980 bp upstream		
15	57999461	Atad2	rs263787034	T	T	C	983 bp upstream		
15	57999461	Atad2	rs263787034	T	T	C	983 bp upstream		
15	57999470	Atad2	rs217611255	G	G	A	992 bp upstream		
15	57999470	Atad2	rs217611255	G	G	A	992 bp upstream		
15	58002908	Ntaq1	rs253370514	A	A	G	1865 bp upstream		
15	58002988	Ntaq1	rs221463437	A	A	G	1785 bp upstream		
15	58003053	Ntaq1	rs253065924	C	C	T	1720 bp upstream		
15	58003234	Ntaq1	rs231271882	C	C	T	1539 bp upstream		
15	58003330	Ntaq1	rs228829024	T	T	C	1443 bp upstream		
15	58003356	Ntaq1	rs249084551	T	T	C	1417 bp upstream		
15	58003369	Ntaq1	rs215459354	G	G	A	1404 bp upstream		
15	58003643	Ntaq1	rs213272817	C	C	G	1130 bp upstream		
15	58003901	Ntaq1	rs244146807	G	G	A	872 bp upstream		
15	58004063	Ntaq1	rs250970630	A	A	C	710 bp upstream		
15	58004076	Ntaq1	rs265024855	A	A	T	697 bp upstream		
15	58004138	Ntaq1	rs212516740	T	T	C	635 bp upstream		
15	58004190	Ntaq1	rs233503292	A	A	G	583 bp upstream		
15	58004345	Ntaq1	rs222753224	T	T	C	428 bp upstream		
15	58004834	Ntaq1	rs220792069	C	C	T	mRNA-UTR		
15	58004852	Ntaq1	rs245306313	G	G	A	mRNA-UTR		
15	58261156	Anxa13	rs224934264	A	A	G	63 bp upstream		
15	58262740	Anxa13	rs230593697	A	A	T	1647 bp upstream		
15	58262751	Anxa13	rs254041111	T	T	C	1658 bp upstream		
15	58262814	Anxa13	rs226822553	G	G	A	1721 bp upstream		
15	58263059	Anxa13	rs230563427	T	T	C	1966 bp upstream		
15	58695261	Tmem65	rs261749968	A	T	A	mRNA-UTR		
15	58953657	Mtss1	rs214424810	A	G	A	mRNA-UTR		

Analysis was performed using the Mouse Genomes Project database. Functional candidate genes are denoted in bold. SIFT (Sorting Intolerant From Tolerant) scores were retrieved from Ensembl.

## Data Availability

All data reported in this article are included in Supplementary Materials.

## Supplementary Materials

The following supporting information can be downloaded at: https://www.mdpi.com/article/10.3390/ijms27198762/s1. All data, including genotyping and phenotypical data, used in this study are included in this excel file.
